# The Danubian cryptic invader *Theodoxus fluviatilis* (Gastropoda: Neritidae) in the River Rhine: a potential indicator for metal pollution?

**DOI:** 10.1007/s10646-021-02485-4

**Published:** 2021-10-08

**Authors:** Louisa Marie Rothmeier, Andreas Martens, Burkard Watermann, Karsten Grabow, Jennifer Bartz, René Sahm

**Affiliations:** 1 Institute for Biology, University of Education, Bismarckstraße 10, 76133 Karlsruhe, Germany; 2 LimnoMar Laboratory for Freshwater and Marine Research, Duvenwischen 4, 22359 Hamburg, Germany; 3 German Environment Agency, Schichauweg 58, 12307 Berlin, Germany

**Keywords:** Chromium, Histopathological alterations, Population parameters, Gonads, River pollution

## Abstract

Metal pollution poses a major threat to aquatic systems especially in anthropogenic influenced areas, in as much as metals are persistent in the environment. The freshwater snail *Theodoxus fluviatilis* has often been used as an indicator species for the ecological status in river monitoring. In the River Rhine, the native Northern-European form of *T. fluviatilis* is nowadays extinct, whilst the Danubian form is spreading along the river. The aim of our study was to investigate if the cryptic invader is affected by metal exposure present in the River Rhine and to discuss its potential as an indicator for metal pollution. Several environmental abiotic (14 water environmental variables plus five common metal concentrations in water and biofilm) and biotic parameters (biofilm mass) were measured across 23 sites along the River Rhine. Five population and six histopathological parameters were evaluated on snails collected at all 23 sites. Aqueous chromium concentration was positively correlated to the damage of male reproductive organs of *T. fluviatilis*, and higher ammonium concentration was correlated to a decrease in snail size and an increase in the proportion of juveniles. None of the analysed snail parameters was negatively correlated to concentrations of other metals measured, like copper and zinc. Therefore, based on the parameters evaluated, our results indicate that the Danubian form of *T. fluviatilis* is only restrictedly suitable as an indicator for metal pollution in the River Rhine system. Further field and laboratory investigations including other stressors are necessary to evaluate the indicator potential of the cryptic invader holistically.

## Introduction

Metal pollution is an important factor affecting the health and safety of aquatic environments (Van Ginneken et al. 2017). Metals like copper, zinc, chromium, and cadmium enter surface waters due to anthropogenic use in many applications, e.g. in mining and metal refining industries. Metal ions are present in dissolved form or adsorbed to suspended matter, able to accumulate in sediments and biofilms, and not biodegradable (Morin et al. 2008; Walker et al. 2012). Although essential for organisms in trace quantities, they can be toxic to aquatic organisms even at low concentrations (µg/L; Van Ginneken et al. 2017; Walker et al. 2012), with an enormous variability of effects across metals and taxa (reviewed by Rainbow 2002).

The catchment area of the River Rhine is massively influenced by anthropogenic activities and pollution, due to its dense population and heavy industrialisation (Cioc 2002; Leuven et al. 2009; Uehlinger et al. 2009). Consequently, inorganic and organic pollutants emitted into the river ecosystem are multiple (e.g., metals, agrochemicals, nutrients, pharmaceuticals, surface paintings), both within point sources like sewage treatment plants, effluents of cooling systems, and industrial wastewater, as well as diffuse sources like land use, agricultural and urban runoff, and atmospheric deposition (Uehlinger et al. 2009). Given its intensive anthropogenic exploitation along with the resulting environmental contamination, the River Rhine is one of the most extensively monitored rivers in the world, with water quality being permanently surveyed by bordering countries and analyses published in public reports (as by the International Commission for the Protection of the Rhine, IKSR/CIPR/ICBR). Compared to the 1970s, the ecological status of the river improved due to comprehensive rehabilitation efforts including the improvement of water quality, restoration of riverine ecosystems and habitat connectivity (Leuven et al. 2009). Though, the current water quality of the River Rhine still does not meet the surface water quality standards as set by the Water Framework Directive, and metals still play an important role in river pollution, with differences at single locations (EU 2000; IKSR/CIPR/ICBR 2018; Sjerps et al. 2017). Hence, metal pollution in the River Rhine is still of importance on the spatial scale, also including areas beside the main stream of the river like industrial areas and harbours which are supposed to be a source for anthropogenic metal pollution (Daehne et al. 2017). However, even if effects of metals towards biota can be assumed at low concentration levels (Rainbow 2002; Walker et al. 2012), to our knowledge a multivariate field study investigating the potential importance of metals is missing, at least for the situation of the River Rhine.

Freshwater snails have often been reported as suitable indicators of metal pollution, since they are abundant in many types of freshwaters, have limited mobility and can accumulate large quantities of metals in their tissues (Das and Khangarot 2011; Dhiman and Pant 2021; Mahmoud and Abu Taleb 2013; Otludil and Ayaz 2020). The freshwater snail *Theodoxus fluviatilis* (Linné, 1758) is native to all large rivers in Central Europe (Bunje 2005; Zettler 2008) and prefers habitats with high oxygen and calcium contents and low salinity (Kangas and Skoog 1978; Zettler et al. 2004). Native populations of the Northern-European form of *T. fluviatilis* disappeared from the River Rhine in the late 1990s for unknown reasons (Westermann et al. 2007), and since 2006, specimens of *T. fluviatilis* phylogenetically originating from populations in the Danube and Black Sea drainages are found in the river (Bunje 2005; Gergs et al. 2015). The Danubian form of *T. fluviatilis* was denoted as cryptic invader (originally defined by Novak 2011), because it has presumably been introduced to the River Rhine by shipping through the Main-Danube canal (Gergs et al. 2015), as shown for several non-indigenous species originating from the Ponto-Caspian region (Alt et al. 2019; Bij de Vaate et al. 2002). It is observed to spread along the River Rhine since its discovery and to establish high population densities even in anthropogenically degraded habitats like industrial harbours (IKSR/CIPR/ICBR 2012; Rothmeier and Martens 2019). Whereas the Northern-European form of the snail has often been used as an indicator organism for the ecological status of the River Rhine, the suitability of the Danubian cryptic invader as indicator for environmental pollution in the River Rhine and possible effects on this particular form on the population and physiological level have not yet been evaluated.

The aim of our study is to examine if the Danubian form of *T. fluviatilis* is affected by metal exposure in the Upper River Rhine and to discuss its potential as indicator organism to detect adverse effects of metal pollution in the River Rhine. Resulting from its feeding behaviour almost exclusively on biofilms (Neumann 1961), *T. fluviatilis* is exposed to metals through the dietary pathway by grazing on contaminated biofilms and through aqueous exposure via gill respiration. Considering this and actual metal pollution in the River Rhine, negative effects on population and physiological parameters of the snail are hypothesised. We measured several abiotic (14 water environmental variables plus water and biofilm concentrations of the five common metals Cu, Cr, Sr, Zn, and Fe), and biotic parameters (biofilm mass) at 23 sites at the German Upper River Rhine. Five population and six histopathological parameters were examined on snails collected at all sites, as histopathology is an effective and suitable tool to analyse the effects of contaminants on aquatic organisms on the physiological level especially under field conditions (Otludil and Ayaz 2020).

## Methods and Materials

### Study sites and measured environmental parameters

**Fig. 1:**
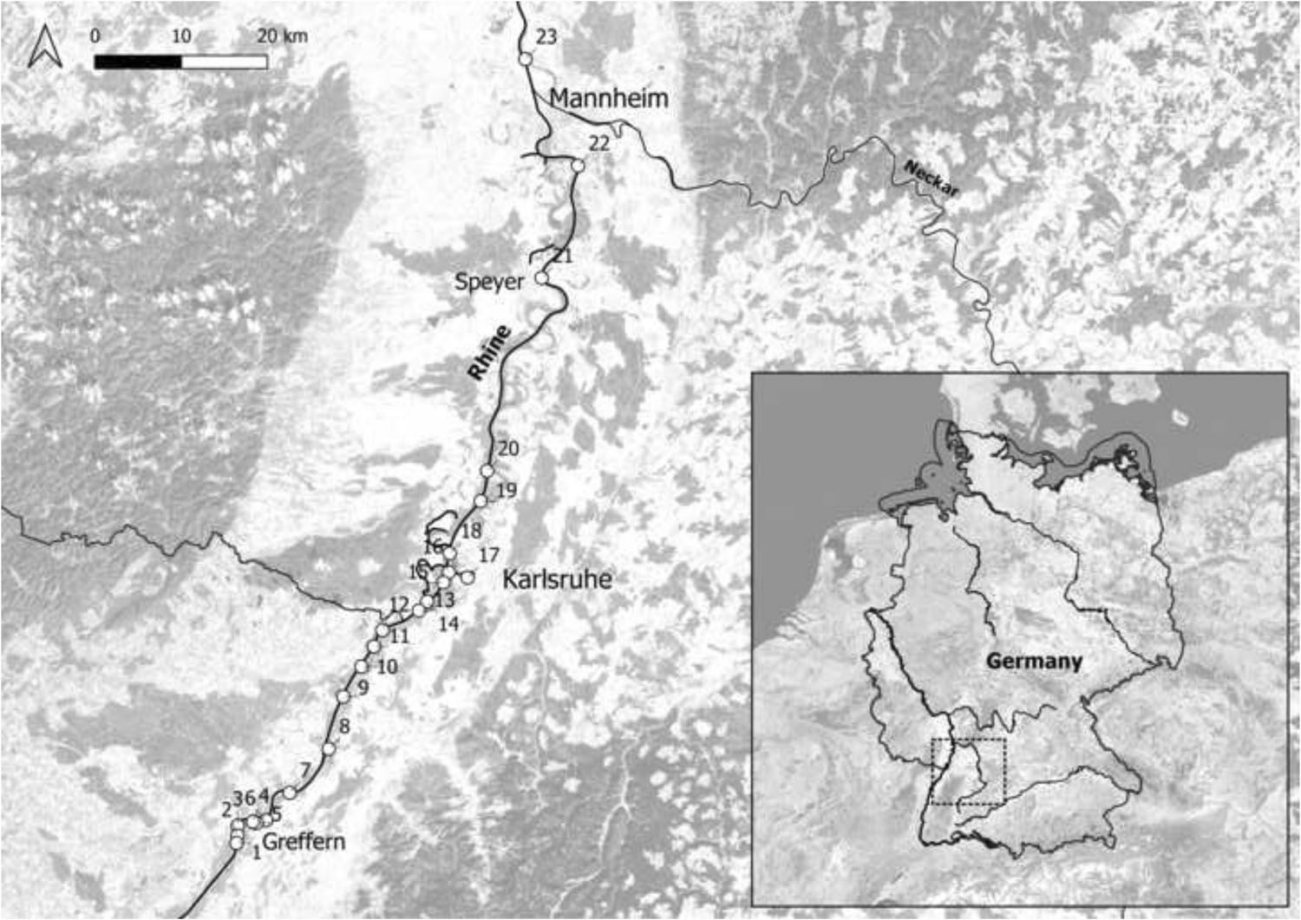
Map of study area showing Germany and its bigger 1st order rivers in overview (large-scale map) and the 23 sampling sites at the Upper River Rhine (detail map). Sampling locations are marked by dots with respective site numbers (for coordinates and further details see Table S1). Map created with the program QGIS (QGIS Development Team 2020)

We sampled 23 sites (S1-S23) located at the German Upper River Rhine, covering 116 km from river-km 316 to 432 (Fig. 1) from August to September 2018 and 2019, respectively. Sites were selected to presumably differ in their metal exposure conditions, being thus situated in different localities of the River Rhine (e.g., close to tributaries, ferry ports, or barrages, within industrial harbours and marinas). Sampling sites at the different localities were chosen to be comparable with respect to visual environmental characteristics (i.e., substrate, occurrence of aquatic macrophytes, water depth) to minimise their potential impact (for further site details see Table S1 in supplementary). At each site, 25 abiotic and biotic environmental parameters were measured (Table 1). Water temperature, dissolved oxygen, pH, and electrical conductivity (using DO-100, DO-100, PH-100 ATC and LWT-01, respectively, Voltcraft, Switzerland) were measured directly in the field. Water samples (1 L) were transported to the laboratory in polypropylene bottles. A subsample of 250 ml was frozen at −22 °C for nutrient measurements until further processing. Nutrients (chloride [Cl^−^], nitrite [NO_2_^–^-N], nitrate [NO_3_^–^-N], phosphate [PO_4_^3–^-P], sulphate [SO_4_^2–^], ammonium [NH_4_^+^-N], calcium [Ca^2+^], magnesium [Mg^2+^], sodium [Na^+^], and silicium [Si]) were analysed from 0.45 µm filtered water samples using continuous-flow-analysis (SAN++, Skalar, The Netherlands) and ion chromatography (Metrohm, Switzerland). Water subsamples for metal analyses (total volume 40 ml) were filtered (0.45 µm) and acidified with ultrapure nitric acid (Merck, Germany) to a final concentration of 0.5% until analysis. At each site, a sample of biofilm was scraped from a defined stone surface with a spatula, stored in river water (total volume 10 ml) and also acidified (ultrapure nitric acid, Merck, Germany, final concentration of 0.5%). Water and biofilm concentrations of five metals (copper [Cu], chromium [Cr], zinc [Zn], strontium [Sr], and iron [Fe]) were quantified using inductively coupled plasma optical emission spectrometry (Perkin Elmer, USA; for details on analytical methods see Rothmeier et al. 2020). To determine periphyton biomass, five subsamples per site were filtered on precombusted glass-fibre filters (Whatman GF6, Ø 25 mm, Maidstone, UK) for ash-free dry mass (AFDM) and dried at 105 °C for 24 h. After weighing (dry mass), the filters were combusted at 550 °C for 8 h and weighed again; the AFDM was calculated by subtraction.

**Table 1:** Environmental parameters measured at the 23 sampling sites of the German Upper River Rhine and their respective measured minimum-maximum ranges

Compartment	Environmental variable	Unit	Range
Water	Temperature	°C	18.2–26.1
	pH	–	7.7–9.0
	Conductivity	µS cm^−1^	230–531
	Dissolved oxygen	mg L^−^^1^	6.0 –10.4
	Chloride (Cl^−^)	mg L^−^^1^	12–39
	Nitrite (NO_2_^−^-N)	mg L^−^^1^	0.005–0.013
	Nitrate (NO_3_^−^-N)	mg L^−^^1^	0.4–1.6
	Phosphate (PO_4_^3−^-P)	mg L^−^^1^	0.001–0.024
	Sulphate (SO_4_^2^^−^)	mg L^−^^1^	21–84
	Ammonium (NH_4_^+^-N)	mg L^−^^1^	0.006–0.030
	Calcium (Ca^2+^)	mg L^−^^1^	25–72
	Magnesium (Mg^2+^)	mg L^−^^1^	6.2–16.3
	Sodium (Na^+^)	mg L^−^^1^	5.0–29.8
	Silicium (Si)	mg L^−^1	0.3–2.8
	Copper (Cu)	µg L^−1^	1.2–9.5
	Chromium (Cr)	µg L^−^^1^	0.8–3.6
	Strontium (Sr)	µg L^−^^1^	213–476
	Zinc (Zn)	µg L^−1^	2.1–17.6
	Iron (Fe)	µg L^−^^1^	34–455
Biofilm	Copper (Cu)	mg kg^−1^	5.8–28.6
	Chromium (Cr)	mg kg^−^^1^	7.4–40.8
	Strontium (Sr)	mg kg^−^^1^	96–570
	Zinc (Zn)	mg kg^−1^	31–155
	Iron (Fe)	mg kg^−^^1^	4,823–14,259
	Biomass of periphyton (AFDM)	mg cm^−2^	0.2–3.7

For detailed measurement values see Supplementary Table S2

### Sampling of *T. fluviatilis* and measured biological parameters

To assess *T. fluviatilis* biological variables, ten randomly chosen riprap stones were collected at each site, for sampling of snails attached to the surface (total n = 1.650 snails; range at sites 11–314 snails: large range due to extremely small (< 20) numbers at S3, S4, S8, S15, and S16, and an extremely large (> 300) number at S11). Five population (snails and egg density, shell size, and female and juvenile percentage) and six histopathological parameters (haemocyte infiltration, midgut gland, kidney and gill dilatation, and female and male gonads pathology) were analysed (Table 2) to determine the individual and population physiological condition of *T. fluviatilis* across sites. To estimate mean snail (individuals m^−2^) and egg (eggs m^−2^) density, the number of snails and egg capsules on their shells was first counted. Then, to estimate stones surface area, stones were wrapped in aluminium foil: by weighing reference aluminium foil pieces, the function of foil weight to corresponding surface value was calculated. Mean snail and egg density were calculated from the ten sampled stones per site (Table 2). Shell length of each individual was measured with a digital calliper (L826.1, Roth, Germany) to the nearest 0.1 mm to calculate mean shell size at sites from all sampled individuals (Table 2). Based on expert judgement (B. Waterman, personal communication), snails with a maximum shell length of 4 mm were considered as premature, in order to calculate the proportion of juvenile snails per site from all individuals (Table 2). A subsample of 13–46 adult snails per site (depending on sufficient availability of snails with shell length > 4 mm) was transferred into bottles with river water from the respective site and transported to the laboratory for histopathological analysis (in total 543 specimens; methodology see Rothmeier et al. 2020). Examination included determination of sex to calculate the proportion of females per site and analysis of the proportion of snails per site affected by pathological findings in tissues and organs, which were haemocyte infiltrations, midgut gland, kidney, or gill dilatations, as well as male gonad pathology (i.e., dilatations in male gonads or accessory glands, and stopped spermatogenesis) and female gonad pathology (i.e., dilatations in female gonads or accessory glands, and stopped oogenesis) (Table 2; Rothmeier et al. 2020). These tissues and organs are commonly investigated in histopathology, because a magnitude of studies indicate pathological changes and/or deformations as responses to a variety of environmental stressors (e.g., Otludil and Ayaz 2020; Rothmeier et al. 2020; Watermann et al. 2008).

**Table 2:** Population and histopathological parameters of the Danubian form of *Theodoxus fluviatilis* (total *n* = 1.650) collected at the 23 sampling sites of the German Upper River Rhine and their respective measured minimum-maximum ranges

	Biological variable	Unit	Range
Population	Mean density	ind m^−2^	8–546
	Mean shell size	mm	3.5–7.5
	Mean density of eggs	eggs m^−2^	0–558
	Female individuals	%	16.7–77.3
	Juvenile individuals	%	4.6–78.3
Histopathology	Haemocyte infiltration	%	0–25
	Midgut gland dilatation	%	0–50
	Kidney dilatation	%	0–6.5
	Gill dilatation	%	0–16.7
	Males: gonads pathology	%	0–25
	Females: gonads pathology	%	0–89

For detailed measurement values see Supplementary Table S2

### Data analysis

Water metal concentrations which were lower than the limit of quantification (LOQ) were calculated as LOQ/2 (Clarke 1998) and those which were lower than the limit of detection (LOD) were taken as zero values (LOQ/LOD: Cu 2.2/0.7 µg/L, Cr 0.7/0.2 µg/L, Sr 0.02/0.01 µg/L, Zn 1.1/0.3 µg/L, Fe 0.8/0.2 µg/L). Abiotic and biotic environmental (Table 1) and biological (population and histopathological) variables of snails (Table 2) were standardised to zero mean and unit variance for dimensionally heterogenous variables. To improve their distribution, the skewed and widespread environmental variables were log(x) transformed prior to analysis (Borcard et al. 2018). Biological *T. fluviatilis* variables were Hellinger-transformed to give low weights to variables with low counts and many zeros (Legendre and Gallagher 2001).

Distance-based redundancy analysis (db-RDA), a constrained ordination method which allows to calculate a dissimilarity matrix of every distance measure (Legendre and Anderson 1999), was used to examine the effects of the abiotic and biotic environmental variables on the five population and six histopathological parameters of *T. fluviatilis*. Since biological *T. fluviatilis* variables contained values of zero in egg density and histopathological variables, Bray-Curtis dissimilarity was used as distance measure. The environmental variables’ variance inflation factors (VIFs) were computed, as strong linear dependencies (auto-correlations) are possible in a large set of explanatory variables. As all environmental variables showed VIF > 10, which indicates strong collinearity (Borcard et al. 2018), a variable selection procedure was conducted: the function *bioenv()* of the R package vegan (version 2.5–7, Oksanen et al. 2020) was used to select the subset of scaled environmental variables whose Euclidean distances had the maximum (rank) correlation to the response dissimilarity matrix (as suggested by Clarke and Ainsworth 1993). Significance of the db-RDA results was analysed by permutation tests using the function *anova()* of vegan (999 permutations). Finally, the univariate approach of Generalised Linear Models (GLM, error distribution = Gaussian) was used to determine the significance of the relationships between single *T. fluviatilis* biological parameters and selected environmental variables. All calculations and statistical analyses were conducted with the program R (version 3.6.3, R Core Team 2020).

## Results

### General description of the environmental and biological measured parameters

At all of the 23 examined sites, concentrations of the five analysed metals (Cu, Cr, Sr, Zn, and Fe) were measured in filtered water and biofilm samples above the LOD. Highest aqueous metal concentrations were found at sites in the main stream, for copper near the city of Grauelsbaum (S1), for chromium, zinc, and iron near the city of Speyer (S21), and for strontium near the city of Mannheim (S23; Table S2). Measured metal concentrations in biofilm samples were highest in the industrial harbour of Karlsruhe for chromium and zinc (S17) and in the main stream near the cities of Söllingen for strontium (S7), Steinmauern for iron (S10), and Speyer for copper (S21; Table S2). The highest concentrations of chloride, nitrate, phosphate, sulphate, calcium, magnesium, and sodium were measured near the city of Mannheim (S23; Table S2). The lowest nitrite concentrations were found at the sites near the cities of Grauelsbaum and Rheinmünster (S1, S4, and S5), and the highest near the city of Altrip (S22; Table S2). At the sampling sites near the cities of Neuburg, Karlsruhe, and Eggenstein (S14, S18, and S19), the lowest ammonium concentrations were measured, whereas the highest ammonium concentration was measured near the lock at Iffezheim (S8; Table S2).

Specimens of the Danubian form of *T. fluviatilis* were present at all sampling sites, showing densities with up to 546 individuals (ind) m^−2^, with densities ranging from 8 ind m^−2^ at a ferry pier near the city of Greffern (S3) to 546 ind m^−2^ in the main stream near the city of Illingen (S11; Table S2). At 22% (5 out of 23) of the examined sites, no egg capsules of *T. fluviatilis* were found on the snail’s shells, whereby highest density of eggs was found in a pleasure boat marina near the city of Rheinmünster (S5, 588 eggs m^−2^; Table S2). The proportion of juvenile snails was lowest in the aforementioned pleasure boat marina (4.6%, S5), with showing a wide range over sampling sites up to 78.3% near the city of Illingen (S11; Table S2). Histopathologic alterations in organs of *T. fluviatilis* were found at all of the examined sites. Most frequent findings were pathologic alterations of female reproductive organs (gonads and accessory glands) at 70% (16 out of 23) of sites and dilatations in midgut glands at 65% (15 out of 23) of sites. Less frequent were pathologic dilatations of snail’s gills at 4% (1 out of 23) of sites and kidneys at 13% (3 out of 23) of sites.

### Correlation between snail and environmental parameters

**Fig. 2:**
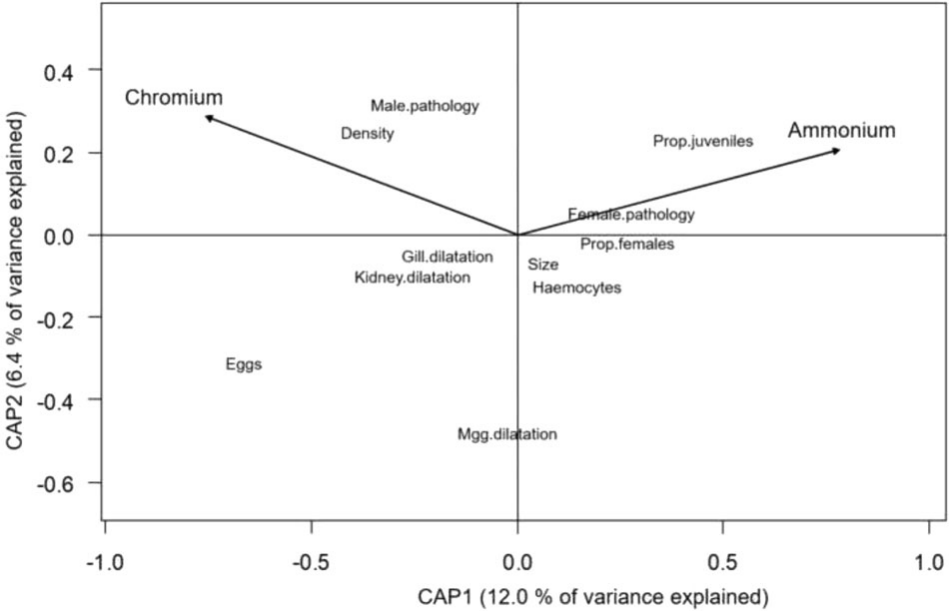
Distance-based redundancy analysis (db-RDA) correlation biplot of the first two canonical axes of *T. fluviatilis* (Danubian form) population and histopathological variables (see Table 2) after *bioenv()* selection of the constraining environmental variables ammonium and chromium concentrations in water (see Table 3). Site scores (*n* = 23) not displayed for clarity, scaling 2 biplot. Ordination based on Bray-Curtis dissimilarity and Hellinger-transformed *T. fluviatilis* biological variables. Abbreviations of constrained *T. fluviatilis* biological variables: Density = mean snail density (ind m^-2^), Eggs = mean egg density (eggs m^-2^), Female.pathology = dilatations in female gonads or accessory glands, and stopped oogenesis, Gill.dilatation = pathologic damage of gill tissue, Haemocytes = pathologic haemocyte infiltrations in tissues, Kidney.dilatation = pathologic damage of kidney tissue, Male.pathology = dilatations in male gonads or accessory glands, and stopped spermatogenesis, Mgg.dilatation = pathologic damage of midgut gland tissue, Prop.females = proportion of female snails, Prop.juveniles = proportion of juvenile snails, Size = mean shell size (mm)

The variable selection procedure showed that water concentrations of ammonium and chromium were the most influential environmental variables in correlation to *T. fluviatilis* biological parameters, based on the parameters evaluated. The db-RDA model of *T. fluviatilis* data constrained by these two explanatory variables showed statistical significance of the global canonical relationship without collinearity of variables (*p*_global model_ < 0.01, *R*^2^_adj_ = 10.2; Fig. 2). All other biotic and abiotic environmental variables showed no significant correlation to the biological snail parameters.

**Fig. 3:**
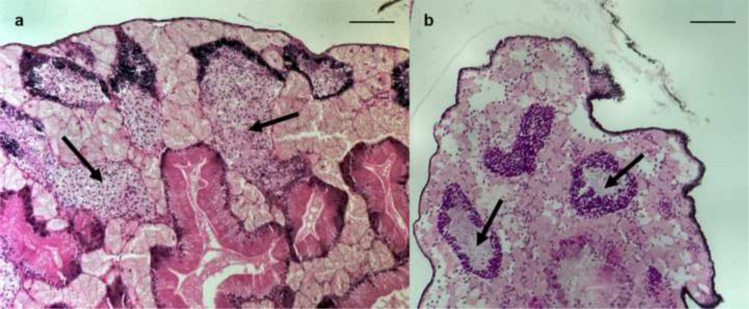
Microscope photographs of histologically prepared sections (2–3 μm) from tissues of the Danubian form of the freshwater snail *Theodoxus fluviatilis* from the River Rhine. Counter-staining with haematoxylin and eosin, bar 50 μm. Photographs showing male snail testis with **a** resorption of spermatogoniae (arrows), **b** arrested ( = stopped) spermatogenesis (arrows)

In the parsimonious db-RDA model with the selected variables, aqueous ammonium concentration had a statistically significant relationship to biological *T. fluviatilis* variables (*p* = 0.01; Table 3a). The proportion of juvenile snails was significantly higher and the mean snail shell size significantly smaller at higher ammonium concentrations (GLM, *p* < 0.01 and < 0.05, respectively; Table 3b). Pathologic alterations in gonads of male snails and impairment of spermatogenesis were significantly more often found at higher aqueous chromium concentrations (GLM, *p* < 0.01; Table 3b, Fig. 3).

**Table 3:** **a** Results of permutation tests (999 permutations) of parsimonious distance-based RDA model after *bioenv()* selection of the two variables ammonium and chromium water concentration constraining five population and six histopathological variables of *T. fluviatilis* (Danubian form). Variance inflation factors (VIF) < 10 show no collinearity of variables. **b** Results of Generalised Linear Model (GLM) analysis showing relationships between ammonium and chromium water concentrations and single *T*. *fluviatilis* population and histopathological variables

Selected environmental variables	Ammonium			Chromium		
**a Distance-based RDA**						
Anova (999 permutations)	F	*p*-value	VIF	*F*	*p*-value	VIF
	2.85	**0.01**	2.94	1.66	0.09	2.94
**b Generalised Linear Model**						
*T. fluviatilis* biological variables	coeff	*p*-value		coeff	*p*-value	
Mean density (ind m^−2^)	−2904	0.43		28	0.26	
Mean shell size (mm)	−65	**0.03**		0.13	0.52	
Mean no. of eggs (eggs m^−2^)	−6195	0.12		18	0.51	
Female individuals (%)	526	0.23		−2.1	0.49	
Juvenile individuals (%)	1547	**0.009**		−4.6	0.28	
Haemocyte infiltration (%)	79	0.63		−1.2	0.26	
Midgut gland dilatation (%)	−355	0.37		−1.5	0.58	
Kidney dilatation (%)	−108	0.05		0.52	0.16	
Gill dilatation (%)	−165	0.11		1.01	0.15	
Males: gonads pathology (%)	−316	0.21		4.9	**0.002**	
Females: gonads pathology (%)	−59	0.94		−2.8	0.59	

Bold values indicate significant effects (*p* < 0.05).

## Discussion

Our hypothesis presuming negative effects of metal pollution on the Danubian form of *T. fluviatilis* in the German Upper River Rhine can only be partly confirmed. According to our results based on the parameters evaluated, the introduced cryptic invader is significantly affected by two factors: exposure towards aqueous chromium and ammonium concentrations. Thus, none of the analysed population and histopathological parameters of Danubian *T. fluviatilis* in our study was negatively affected by any of the measured metal concentrations in water and biofilm except chromium. However, as metal speciation can be an important criterion to analyse the toxicity of metals for aquatic biota (Di Toro et al. 2001; Santore et al. 2001), and considering the fact that water parameters in our study were exclusively measured from filtered water samples, our results are limited to the specification of our study design. Hence, more investigations also regarding concentrations of metal ions bound to particles are necessary to evaluate effects of metal exposure holistically.

At the examined sites in the Upper Rhine, which were partly close to industry locations, chromium exposure was connected to pathologic alterations in male reproductive organs of Danubian *T. fluviatilis*. Playing an important role in environmental pollution in general due to its wide use in many industries like steel welding, mining, or coating applications (Dhiman 2020; Sivakumar et al. 2014), chromium is one of the more toxic metals for biota. Its toxicity cannot be reduced by protein binding and accumulation within organisms to reduce its bioavailability, therefore it needs to be completely detoxified or excreted (Rainbow 2002; Sivakumar et al. 2014). Moreover, especially hexavalent compounds of chromium (Cr^6+^) have in many laboratory studies shown to be highly toxic for animals, leading to negative effects of chromium exposure on male reproductive systems including morphological damage, altered testicular biochemistry or decreases in testis proteins (summarised by Campbell et al. 2009). Toxicity studies using the terrestrial snail *Helix aspersa* demonstrated acute chromium exposure to cause increased mortality and electrolyte disturbance in test animals (24h-LC_50_ 15.13 mg/L, Dhiman 2020). The findings in our study indicate its toxicity to be of a relevance for aquatic snails as well. Given the fact that the effects of water chromium concentration on snails in our study were of a higher importance compared to the other measured metal concentrations, it is presumed to be a factor which has to be considered for acting upon organisms in the anthropogenically influenced environment of the River Rhine. Dissolved aqueous chromium concentrations measured at sampling sites in our study (0.8−3.6 µg/L, Table 1) are partly higher than concentrations of < 0.2−0.33 µg/L reported by the IKSR/CIPR/ICBR (2018). Although annual chromium concentrations in the River Rhine are significantly lower than the national environmental quality standard for surface waters (640 µg/L, IKSR/CIPR/ICBR 2018), the importance of a continuous surveillance of the metal in the river, keeping in mind the negative effects of chromium concentration on *T. fluviatilis* according to the results of our study, is emphasised.

The positive correlation between higher ammonium concentrations and smaller snail size and higher proportion of juvenile *T. fluviatilis* in this study can presumably be explained by a direct effect. Ammonia is a known degradation product of organic matter and therefore a potential chemical indicator for eutrophication in freshwater systems (Zaghloul et al. 2019), which in turn can lead to higher primary productivity. As *T. fluviatilis* is a nearly exclusive biofilm grazer feeding on diatoms (Neumann 1961), a higher food supply due to a higher amount of the current total biofilm biomass at sites with higher ammonium concentrations is likely to lead to increased reproduction and therefore a higher proportion of smaller and juvenile snails.

Due to their wide use in metal industries or as components of biocides and therefore relevant emission into surface waters, the metals copper and zinc play an important role for environmental pollution (Walker et al. 2012). In a number of European marinas, copper concentrations were too high for the approval of copper-based antifouling paints (Lagerström et al. 2020; Ytreberg et al. 2021). Although copper and zinc are essential trace metals being components of enzymes and proteins in organisms (Rainbow 2002), toxicity occurs when threshold metal concentrations are exceeded, but also in function of the organism, its body mass and metal bioavailability (Fent 2004; Van Ginneken et al. 2017). In general, water concentrations of copper and zinc in the River Rhine are below national environmental quality standards for surface waters (160 µg/L for copper and 800 µg/L for zinc). Though, measured dissolved aqueous copper and zinc concentrations at sampling sites in our study (1.2−9.5 µg/L Cu and 2.1−17.6 µg/L Zn; Table 1) were hypothesised to have a potential negative effect on biological parameters of Danubian *T. fluviatilis*, as they exceed values of comparable studies. They were partly higher than concentrations of the IKSR monitoring (0.77−2.4 µg/L Cu and < 1−5.3 µg/L Zn; IKSR/CIPR/ICBR 2018), which could be due to the fact that sites with a potential higher metal exposure, like marinas or industrial harbours, were selected. Furthermore, both copper and zinc concentrations in our study were at individual sites higher than concentrations found in a comparable study in Swedish harbours leading to higher mortality, reduced growth, and lower fecundity of the brackish water form of *T. fluviatilis* (2.7−3.7 µg/L Cu and 7.1−10.6 µg/L Zn; Bighiu et al. 2017). Given our results showing no significant effects of detected copper and zinc concentrations on population and histopathological parameters of the Danubian form of *T. fluviatilis* in the field, it can be presumed that the cryptic invader is able to cope with concentrations of these metals at the examined sites. This assumption is supported by the fact that, considering copper concentrations, our measurements are lower than the 21-day LC_50_ for copper of 16 µg/L derived from a laboratory study with the Danubian form of *T. fluviatilis* (Rothmeier et al. 2020). Furthermore, the newly introduced snail shows high population densities up to more than 500 individuals m^−2^ (Table 2), with a density of more than 100 individuals m^−2^ at site S21 where aqueous chromium, zinc, and iron concentrations were highest (Table S2). Due to this lack of sensitivity, we conclude that the Danubian form of *T. fluviatilis* is only restrictedly suitable as an indicator considering at least metal pollution in light of the parameters evaluated in our field study in the River Rhine.

However, one must also consider the enormous variability of effects of not only metals, but also other environmental pollutants, across metals and invertebrate taxa. Snails have various detoxification mechanisms to counteract metal toxicity and reduce their bioavailability (Bighiu et al. 2017; Mahmoud and Abu Taleb 2013; Watermann et al. 2008), which is not the case for several other sensitive taxa. The knowledge of effects on a chosen species is essential for environmental monitoring, but concentrations should be compared considering the whole variety of invertebrate species, also taking the transfer of metals along food chains and the community level into account (Rainbow 2002). Furthermore, the difference in sensitivity between indigenous and invading species, as well as intraspecific variability, may play an important role in the future.

## Supplementary Information

Supplementary Materails

Supplementary Materails

## Data Availability

The datasets generated during and/or analysed during the current study are available from the corresponding author on reasonable request.
