# Supplementary material for: The Danubian cryptic invader Theodoxus fluviatilis (Gastropoda: Neritidae) in the River Rhine: a potential indicator for metal pollution?
Source: Ecotoxicology. 2021 Oct 8;31(1):24–32. doi: 10.1007/s10646-021-02485-4 (PMC8752526; doi:10.1007/s10646-021-02485-4)
Supplement: Supplementary file 1 — Supplementary Materails [file 10646_2021_2485_MOESM1_ESM.pdf]

Supplementary material to

The Danubian cryptic invader *Theodoxus fluviatilis* (Gastropoda: Neritidae) in the River Rhine: a potential indicator for metal pollution?

Louisa Marie Rothmeier[1], Andreas Martens[1], Burkard Watermann[2], Karsten Grabow[1], Jennifer Bartz[3], René Sahm[3]

[1] Institute for Biology, University of Education, Bismarckstraße 10, 76133 Karlsruhe, Germany

[2] LimnoMar Laboratory for Freshwater and Marine Research, Duvenwischen 4, 22359 Hamburg, Germany

[3] German Environment Agency, Schichauweg 58, 12307 Berlin, Germany

Corresponding author

Louisa Marie Rothmeier

Institute for Biology

University of Education

Bismarckstraße 10

76133 Karlsruhe

Germany

E-mail address: louisa-rothmeier@gmx.de

Table S1: Sampling sites (number, km, name, and coordinate) and dates of sample collection at the German Upper River Rhine.

No.	Km	Name	Coordinate (WGS84)	Sampling date (month/year)
1	316	Grauelsbaum	48°44'10''N, 7°58'08''E	09/2019
2	317	Grauelsbaum, harbour	48°44'38''N, 7°58'14''E	09/2019
3	318	Greffern, ferry pier	48°45'15''N, 7°58'15''E	09/2019
4	320	Rheinmünster	48°45'29''N, 7°59'41''E	09/2019
5	321	Rheinmünster, marina	48°45'23''N, 8°00'01''E	09/2019
6	322	Rheinmünster, harbour	48°45'35''N, 8°00'56''E	09/2019
7	328	Söllingen	48°47'18''N, 8°03'09''E	09/2019
8	334	Iffezheim, lock	48°50'03''N, 8°06'52''E	09/2019
9	340	Plittersdorf, ferry pier	48°53'16''N, 8°08'14''E	09/2019
10	345	Steinmauern	48°55'10''N, 8°09'56''E	09/2019
11	347	Illingen	48°56'24''N, 8°11'08''E	09/2019
12	349	Au am Rhein	48°57'26''N, 8°11'59''E	09/2019
13	354	Neuburgweier, ferry pier	48°58'37''N, 8°15'22''E	08/2019
14	355	Neuburg	48°59'12''N, 8°16'10''E	09/2018
15	359	Daxlanden	49°00'21''N, 8°17'41''E	08/2019
16	360	Karlsruhe, harbour basin 1	49°00'59''N, 8°18'12''E	08/2019
17	360	Karlsruhe, harbour basin 4	49°00'40''N, 8°19'57''E	08/2019
18	362	Karlsruhe	49°02'07''N, 8°18'16''E	09/2018
19	369	Eggenstein	49°05'24''N, 8°21'13''E	09/2018
20	372	Leimersheim	49°07'17''N, 8°21'50''E	08/2018
21	400	Speyer	49°19'10''N, 8°26'59''E	08/2018
22	414	Altrip	49°26'03''N, 8°30'25''E	08/2018
23	432	Mannheim	49°32'37''N, 8°25'26''E	08/2018

Table S2 (excel table): Detailed measurement values of environmental parameters (Table 1) and *Theodoxus fluviatilis* biological variables (Table 2). Minimum and maximum values of respective variables are marked in bold.
